# Gene expression panel predicts metastatic‐lethal prostate cancer outcomes in men diagnosed with clinically localized prostate cancer

**DOI:** 10.1002/1878-0261.12014

**Published:** 2016-10-19

**Authors:** Rohina Rubicz, Shanshan Zhao, Jonathan L. Wright, Ilsa Coleman, Catherine Grasso, Milan S. Geybels, Amy Leonardson, Suzanne Kolb, Craig April, Marina Bibikova, Dean Troyer, Raymond Lance, Daniel W. Lin, Elaine A. Ostrander, Peter S. Nelson, Jian‐Bing Fan, Ziding Feng, Janet L. Stanford

**Affiliations:** ^1^ Division of Public Health Sciences Fred Hutchinson Cancer Research Center Seattle WA USA; ^2^ Biostatistics and Computational Biology Branch National Institute of Environmental Health Sciences Research Triangle Park Durham NC USA; ^3^ Department of Urology University of Washington School of Medicine Seattle WA USA; ^4^ Division of Human Biology Fred Hutchinson Cancer Research Center Seattle WA USA; ^5^ Department of Epidemiology GROW School for Oncology and Developmental Biology Maastricht University The Netherlands; ^6^ Illumina, Inc. San Diego CA USA; ^7^ Departments of Pathology and Microbiology and Molecular Cell Biology Eastern Virginia Medical School Norfolk VA USA; ^8^ Department of Urology Eastern Virginia Medical School Norfolk VA USA; ^9^ Cancer Genetics and Comparative Genomics Branch National Human Genome Research Institute NIH Bethesda MD USA; ^10^ Division of Clinical Research Fred Hutchinson Cancer Research Center Seattle WA USA; ^11^ Department of Medicine University of Washington School of Medicine Seattle WA USA; ^12^ Department of Biostatistics University of Texas MD Anderson Cancer Center Houston TX USA; ^13^ Department of Epidemiology School of Public Health University of Washington Seattle WA USA; ^14^ Present address: AnchorDx Corp. Guangzhou 510300 China

**Keywords:** biomarkers, gene expression, metastasis, prognosis, prostate cancer, validation

## Abstract

Prognostic biomarkers are needed to distinguish patients with clinically localized prostate cancer (PCa) who are at high risk of metastatic progression. The tumor transcriptome may reveal its aggressiveness potential and have utility for predicting adverse patient outcomes. Genomewide gene expression levels were measured in primary tumor samples of 383 patients in a population‐based discovery cohort, and from an independent clinical validation dataset of 78 patients. Patients were followed for ≥ 5 years after radical prostatectomy to ascertain outcomes. Area under the receiver‐operating characteristic curve (AUC), partial AUC (pAUC, 95% specificity), and *P*‐value criteria were used to detect and validate the differentially expressed transcripts. Twenty‐three differentially expressed transcripts in patients with metastatic‐lethal compared with nonrecurrent PCa were validated (*P *<* *0.05; false discovery rate < 0.20) in the independent dataset. The addition of each validated transcript to a model with Gleason score showed that 17 transcripts significantly improved the AUC (range: 0.83–0.88; all *P‐*values < 0.05). These differentially expressed mRNAs represent genes with diverse cellular functions related to tumor aggressiveness. This study validated 23 gene transcripts for predicting metastatic‐lethal PCa in patients surgically treated for clinically localized disease. Several of these mRNA biomarkers have clinical potential for identifying the subset of PCa patients with more aggressive tumors who would benefit from closer monitoring and adjuvant therapy.

**Abbreviations**
AUC: area under the receiver‐operating characteristic curve
EV: Eastern Virginia
FDR: false discovery rate
FFPE: formalin‐fixed, paraffin‐embedded
FH: Fred Hutchinson
mRNA: messenger RNA
pAUC: partial AUC
PCa: prostate cancer
PSA: prostate‐specific antigen
RNA: ribonucleic acid
RP: radical prostatectomy
SEER: Surveillance, Epidemiology, and End Results

## 1 Introduction

In the United States, an estimated 180 890 new cases of prostate cancer (PCa) will be diagnosed in 2016 and more than 26 000 will die from the disease, making PCa the second leading cause of cancer‐related death among American men (Siegel *et al*., 2016). PCa is biologically heterogeneous and has a variable clinical course; therefore, it is a challenge to predict which patients diagnosed with localized tumors will experience metastatic progression. Gleason score is currently the best clinical variable for determining tumor aggressiveness and metastatic potential. Prognostic biomarkers that can improve upon Gleason score and accurately determine the individualized risk of metastatic progression for men diagnosed with clinically localized PCa and treated with radical prostatectomy (RP) are urgently needed.

Tumor‐derived gene expression signatures have been shown to have predictive power for distinguishing between patients with more aggressive vs. less aggressive PCa (Bostrom *et al*., 2015; Ross *et al*., 2016). These gene expression levels are the product of underlying tumor genomic variation and epigenomic modification, both of which may influence metastatic progression (Alumkal *et al*., 2008; Goering *et al*., 2012; Jeronimo *et al*., 2011; Schoenborn *et al*., 2013). Most reported gene expression panels were selected from a limited set of selected genes or biological pathways previously implicated in carcinogenesis (Cuzick *et al*., 2011; Klein *et al*., 2014; Nakagawa *et al*., 2008; Penney *et al*., 2011). Of the three commercially available gene expression panels for predicting PCa outcomes, only one (Decipher, GenomeDx) was constructed from an analysis of genomewide mRNA data (Erho *et al*., 2013). The Decipher panel uses 22 transcripts to predict clinical metastasis in patients treated with RP. Some gene expression panels have been validated by subsequent studies to inform secondary treatment decisions (Bishoff *et al*., 2014; Cooperberg *et al*., 2013; Klein *et al*., 2016; Knezevic *et al*., 2013). However, there remains substantial room to improve the prognostic capabilities of these expression panels, including identifying other transcripts that capture additional information about tumor aggressiveness.

Here, we examine genomewide tumor gene expression levels in a population‐based cohort of patients with PCa who underwent RP as primary therapy, with the goal of identifying novel prognostic biomarkers. Transcriptome data were used to select the most informative gene transcripts based on their ability to improve upon Gleason score for predicting metastatic progression. Promising transcripts were then analyzed in an independent cohort for validation of their accuracy as prognostic biomarkers.

## 2 Materials and methods

### 2.1 Study population

This study included 383 men of European American ancestry from the Fred Hutchinson (FH) Cancer Research Center cohort who were diagnosed with clinically localized PCa and underwent RP as primary treatment. These patients were previously enrolled in population‐based studies of PCa in residents of King County, Washington (Agalliu *et al*., 2008; Stanford *et al*., 1999). The first study included men aged 40–64 years who were diagnosed between 1993 and 1996, while the second study focused on men aged 35–74 years who were diagnosed between 2002 and 2005. The Fred Hutchinson Institutional Review Board approved the study, and all participants signed informed consent statements. Clinical information including PSA level at diagnosis, pathological tumor stage (local = pT2, N0/NX, M0; regional = pT3–T4 and/or N1, M0), and Gleason score was collected from the Seattle‐Puget Sound Surveillance, Epidemiology, and End Results (SEER) cancer registry. PCa recurrence status was determined from the data collected in two follow‐up surveys that were completed by patients in 2004–2005 and in 2010–2011, with review of medical records and physician follow‐up for clarification as needed. Patients were classified as having PCa recurrence if they reported a postsurgery PSA of ≥ 0.2 ng·mL^−1^, had received secondary treatment (e.g., salvage radiation, androgen deprivation therapy, orchiectomy, or chemotherapy), had a positive lymph node or prostate bed biopsy, MRI, CT, or bone scan showing metastatic PCa, were told by a physician that the PCa had recurred, or died from PCa. Vital status and underlying cause of death were obtained from the SEER cancer registry, and cause of death was confirmed by review of death certificates. Patients who developed metastasis or died of PCa were combined in a metastatic‐lethal category. Over an average follow‐up time of 12.8 years, there were 278 patients who had no evidence of PCa recurrence and 27 patients who progressed to metastatic‐lethal PCa and were included in the analyses.

The validation dataset consisted of 78 European American men diagnosed with clinically localized PCa who had radical prostatectomy and were treated at Eastern Virginia (EV) Medical School. The dataset included 32 men with metastatic or lethal PCa and 46 men with no evidence of recurrence (nested case–control design); these patients were diagnosed and treated during a similar time period as those in the discovery cohort. Metastatic‐lethal PCa was identified using a similar protocol as for the FH cohort. These men were diagnosed with PCa in 1992–2009 and were followed for PCa outcomes on average for 9.0 years.

### 2.2 Sample preparation and RNA extraction

Formalin‐fixed, paraffin‐embedded (FFPE) PCa tumor tissue blocks were obtained from radical prostatectomy samples and used to make H&E‐stained slides, which were reviewed by a PCa pathologist to confirm the presence of and the location of prostate adenocarcinoma. For each patient, two 1‐mm tumor tissue cores were taken from the areas enriched with ≥ 75% tumor cells from the dominant lesion. For 20 patients, benign adjacent tissue cores were also taken. The RNeasy^®^ FFPE Kit (Qiagen Inc., Valencia, CA, USA) was used to isolate the RNA from tissue cores, and the samples were quantified with RiboGreen, aliquoted (200 ng per patient) onto 96‐well plates, and shipped to Illumina for gene expression profiling (April *et al*., 2009). Tumor RNA samples from patients with various outcomes were randomly distributed across the plates and laboratory personnel were blinded to this information.

### 2.3 Gene expression profiling

The WG_DASL^®^ HT Assay (Illumina, Inc., San Diego, CA, USA) was used for gene expression profiling. RNA was reverse‐transcribed to cDNA using biotinylated oligo (dT) and random nonamer primers and immobilized to a streptavidin‐coated solid support. Prequalification of cDNA was assessed using quantitative RT‐PCR and the analysis of housekeeping gene *RPL13a*. Biotinylated cDNAs were annealed to assay‐specific oligonucleotides to create PCR templates that were amplified using labeled and biotinylated universal primers. Labeled PCR products were captured on streptavidin paramagnetic beads, washed, and denatured to yield single‐stranded fluorescent molecules that were hybridized to the HumanHT‐12 v4 Expression BeadChip. Samples were scanned using a BeadArray^®^ Reader that reads the fluorescence intensities, and intensity data file images were extracted for 29 377 transcripts that map to 20 818 genes.

### 2.4 Statistical analysis

Gene expression data were quantile‐normalized and log2‐transformed (R Core Development Team, 2012; http://cran.r-project.org/). Low‐quality probes were filtered out with IlluminaHumanWGDASLv4.db in R Bioconductor, leaving 26 051 transcripts for further analysis. Batch effects were removed using ComBat (Johnson *et al*., 2007). FH blind duplicate samples from 11 patients that were randomly distributed across the plates had correlations ranging from 0.98 to 0.99, and replicate samples from two patients that were included on every plate had mean correlations of 0.99. For the EV cohort, there were blind duplicate samples from eight patients and replicates samples from four patients, all with correlations ≥ 0.99.

The analysis strategy utilized a sequential selection, panel building, and refinement approach (Feng *et al*., 2004). As a first step, the FH discovery cohort was used to assess the ability of each of the 26 051 transcripts to distinguish men with metastatic‐lethal PCa (*n *=* *27) from men with no evidence of recurrence (*n *=* *278). The AUC (area under the receiver‐operating characteristic curve) and pAUC (partial AUC) (Ma *et al*., 2013) were calculated for each transcript. While the AUC evaluates the overall performance, the pAUC can be used to evaluate the performance at a fixed high specificity (or sensitivity). We calculated the pAUC at 95% specificity, aiming to select transcripts with a low false‐positive rate for classifying patients with metastatic‐lethal PCa. This approach increases confidence that patients classified as high risk by the biomarker in fact have high‐risk tumors, which is important if these men are to undergo more aggressive monitoring and treatment regimens. Those transcripts that ranked in the top 4% based on pAUC and the top 1% based on AUC were included in a reduced biomarker panel (*n *=* *1216 transcripts).

We next identified in the FH cohort the subset of biomarkers in the reduced panel of 1216 mRNAs that showed the greatest improvement over Gleason score, because we aimed to find the transcripts that were complimentary to Gleason score in predicting tumor aggressiveness. Other potential prognostic classifiers were also considered, including age at diagnosis, diagnostic PSA level, and pathological tumor stage (local = pT2, N0/NX, M0; regional = pT3–T4 and/or N1, M0); however, these did not improve upon Gleason score alone (*P*‐values for likelihood ratio test > 0.05) and therefore were not included in subsequent models. A logistic regression model was fit with Gleason score as the predictor and metastatic‐lethal vs. nonrecurrent PCa as the outcome. Using that base model, forward model building was performed for transcript selection based on three criteria to judge the model performance: pAUC (at specificity of 0.95), AUC, and *P*‐value (Wald test). Transcript selection continued until the model's increase in pAUC was less than 0.0005, increase in AUC was less than 0.005, or *P*‐value was greater than 0.05. To avoid randomness, for each criterion we bootstrapped random samples 1000 times and repeated the stepwise model building on each bootstrap sample. Those transcripts (*n *=* *48) that appeared more than 40 times in at least one of the 1000 panels based on each of the three criteria were picked as the most robust markers for further analysis.

The panel of 48 robust transcripts identified in the FH cohort was then evaluated in the independent EV testing dataset. For each transcript, the AUC and pAUC (at 95% specificity) were calculated for classifying metastatic‐lethal vs. nonrecurrent PCa. A *t*‐test was also performed for each biomarker to compare the mean mRNA levels between the patient groups. *P*‐values for the AUC and pAUC were computed using 10 000 permutations, and 95% confidence intervals were calculated using 2000 stratified bootstrap replicates (pROC package in r). A *P*‐value threshold of 0.05 (two‐tailed test) was considered statistically significant, and a false discovery rate (FDR) of less than 0.20 was considered noteworthy to account for multiple testing in the validation dataset (Benjamini and Hochberg, 1995). Likelihood ratio tests were also computed to compare the goodness of fit for base models fit with Gleason score alone and alternative models fit with Gleason score plus a transcript. All statistical analyses were conducted using the r statistical computing software (http://cran.r-project.org/).

In order to identify molecular drivers of the genes in the transcript signature, we used the Ingenuity Pathway Analysis (Ingenuity Systems^®^, www.ingenuity.com) to perform Upstream Regulator Analysis (Kramer *et al*., 2014). The analysis was restricted to experimentally observed results from within the Ingenuity Knowledge Base. Only direct relationships with Fisher's exact test overlap *P*‐values < 0.05 were considered in molecular associations. Gene set enrichment analysis (GSEA) was also performed to identify the pathways that were overrepresented by the set of gene transcripts in the validated expression panel (Subramanian *et al*., 2005).

## 3 Results

### 3.1 Patient cohort characteristics

For both the FH and EV patient cohorts, the mean age at diagnosis of patients with metastatic‐lethal PCa was similar to that of patients with no evidence of recurrence (Table 1). As expected, men who progressed to metastatic‐lethal outcomes were more likely to have higher Gleason scores, regional stage disease, and higher diagnostic PSA levels in both cohorts.

**Table 1:** Selected characteristics of the Fred Hutchinson (FH) and Eastern Virginia (EV) prostate cancer patients. SD, standard deviation; PSA, prostate‐specific antigen

	FH training dataset (*n *=* *305)						*P*‐value^a^	EV testing dataset (*n *=* *78)						*P*‐value^a^
	No recurrence			Metastatic‐lethal				No recurrence			Metastatic‐lethal			
	*n *=* *278			*n *=* *27				*n *=* *46			*n *=* *32			
	No.	%	Mean (SD)	No.	%	Mean (SD)		No.	%	Mean (SD)	No.	%	Mean (SD)	
Age at diagnosis (year)			58.2 (6.9)			57.8 (6.7)	0.38			59.8 (6.6)			60.1 (5.9)	0.41
Gleason score														
≤ 6	157	56.5		5	18.5		< 0.01	14	30.4		2	6.2		< 0.01
7(3 + 4)	93	33.4		11	40.7			27	58.7		9	28.1		
7(4 + 3)	15	5.4		5	18.5			2	4.3		7	21.9		
8–10	13	4.7		6	22.2			3	6.5		14	43.8		
Pathological stage^b^														
Local	213	76.6		13	48.1		< 0.01	26	56.5		0	0		< 0.01
Regional	65	23.4		14	51.9			20	43.5		32	100		
PSA (ng·mL^−1^) at diagnosis														
< 4.0	47	16.9		2	7.4		< 0.01	11	23.9		5	15.6		< 0.01
4.0–4.9	174	62.6		7	25.9			30	65.2		19	59.4		
10.0–19.9	30	10.8		6	22.2			3	6.5		6	18.8		
≥ 20	11	4.0		8	29.6			1	2.0		2	6.2		
Missing	16	5.7		4	14.8			1	2.0		0	0		

^a^ A *t*‐test (age) or chi‐square test was used (all categorical variables). ^b^ Local = pT2, N0/NX, M0; Regional = pT3–T4 and/or N1, M0.

### 3.2 Evaluation of gene transcripts in the discovery cohort

The 48 transcripts that were most predictive of metastatic‐lethal PCa events in the FH discovery cohort are included in Table S1. These biomarkers were selected based on their ability to improve the prognostic classification above Gleason score alone. The number of times each biomarker was selected for model inclusion by each criterion (AUC, pAUC, and *P*‐value) is shown in Table S2. Approximately half of the transcripts (25 of 48) had higher mRNA expression levels in metastatic‐lethal PCa patients compared with patients without recurrence, and the fold change in mRNA levels between the two patient groups ranged from 0.39 to 2.55 (Table S1). The AUC and pAUC values for predicting metastatic‐lethal PCa events ranged from 0.54 to 0.84 and 0.0036 to 0.0186, respectively.

### 3.3 Evaluation of gene transcripts in the validation dataset

The panel of 48 top‐ranked transcripts identified through a sequential selection strategy in the training dataset was then evaluated in the EV testing dataset. Forty‐one (85.4%) of the transcripts had gene expression differences in the same direction (e.g., overexpression in metastatic‐lethal compared with nonrecurrent patients) in the EV cohort as was observed in the FH training cohort. Twenty‐three (47.9%) of the 48 transcripts were validated based on significant AUC, pAUC, and/or *t*‐test *P*‐values (Table 2); all FDRs < 0.20 (Table 2). Of these, ten transcripts were significant based on all three criteria, including those for genes *ALDH1A2, CLTCL1*,* DPT*,* ITGA11*,* KLC3*,* PNMAL1*,* SPRY4*,* TNFSF4*,* TSC22D3*, and *ZNF704*. The transcript with the highest AUC was for *SRD5A2* (AUC = 0.78, *P*‐value = 1.0 × 10^−4^); mRNA expression differences for *ALDH1A2* and *TSC22D3* were the most significant according to the AUC criterion (*P*‐values < 1.00 × 10^−6^). The transcript with the highest pAUC was for gene *KLC3* (pAUC = 0.0154, *P*‐value < 1.0 × 10^−6^). The differential expression *t*‐test *P*‐value was < 0.0008 for five transcripts (for genes *ALDH1A2, CLTCL1*,* SRD5A2, TSC22D3*, and *ZNF704*). A heat map of the 23 validated transcripts in the two patient groups (metastatic‐lethal and nonrecurrence) is included in Fig. S1.

**Table 2:** Validated transcripts (*n *=* *23) for classifying metastatic‐lethal vs. nonrecurrent prostate cancer in the Eastern Virginia testing dataset. Significant *P*‐values are shown in boldface

Transcript ID	Gene	Chr.	Mean expression nonrecurrence	Mean expression metastatic‐lethal	Difference in mean expression	Fold change	AUC	*P*‐value AUC	pAUC	*P*‐value pAUC	*P*‐value *t*‐test^a^	FDR *Q*‐value
ILMN_1748538	*ALDH1A2*	15	11.44	10.39	−1.05	0.48	0.75	**< 1.00 × 10** ^**−6**^	0.0135	**5.00 × 10** ^**−4**^	**1.60 × 10** ^**−4**^	3.09 × 10^−3^
ILMN_1786125	*CCNA2*	4	10.99	11.29	0.30	1.23	0.61	8.42 × 10^−2^	0.0078	**1.96 × 10** ^**−2**^	6.91 × 10^−2^	1.58 × 10^−1^
ILMN_1716279	*CENPE*	4	7.93	8.27	0.34	1.26	0.62	7.66 × 10^−2^	0.0046	0.144	**4.42 × 10** ^**−2**^	1.31 × 10^−1^
ILMN_1694584	*CLTCL1*	22	7.84	8.44	0.60	1.52	0.72	**8.00 × 10** ^**−4**^	0.0089	**1.12 × 10** ^**−2**^	**7.85 × 10** ^**−4**^	7.53 × 10^−3^
ILMN_1673843	*CST2*	20	8.71	9.37	0.66	1.58	0.65	**2.94 × 10** ^**−2**^	0.0029	0.326	5.19 × 10^−2^	1.33 × 10^−1^
ILMN_1708107	*DPT*	1	10.74	9.85	−0.89	0.54	0.70	**3.00 × 10** ^**−3**^	0.0066	**4.68 × 10** ^**−2**^	**4.10 × 10** ^**−3**^	2.46 × 10^−2^
ILMN_1700541	*FBLN1*	22	13.81	13.30	−0.51	0.70	0.72	**1.30 × 10** ^**−3**^	0.0051	9.39 × 10^−2^	**2.49 × 10** ^**−3**^	1.70 × 10^−2^
ILMN_1756358	*FBXO36*	2	11.20	11.50	0.31	1.24	0.72	**6.00 × 10** ^**−4**^	0.0010	0.678	8.79 × 10^−2^	1.83 × 10^−1^
ILMN_2406084	*ITGA11*	15	9.56	10.24	0.68	1.61	0.69	**5.00 × 10** ^**−3**^	0.0075	**2.55 × 10** ^**−2**^	**1.10 × 10** ^**−2**^	5.84 × 10^−2^
ILMN_1702738	*KLC3*	19	7.32	7.67	0.35	1.27	0.67	**1.18 × 10** ^**−2**^	0.0154	**< 1.00 × 10** ^**−6**^	**2.23 × 10** ^**−3**^	1.70 × 10^−2^
ILMN_1661895	*PI15*	8	12.48	11.99	−0.49	0.71	0.68	**6.50 × 10** ^**−3**^	0.0002	0.808	**3.16 × 10** ^**−2**^	1.08 × 10^−1^
ILMN_1734810	*PJA1*	X	9.40	9.06	−0.34	0.79	0.64	**4.08 × 10** ^**−2**^	0.0051	9.63 × 10^−2^	5.26 × 10^−2^	1.33 × 10^−1^
ILMN_1737025	*PLCL2*	3	10.45	9.92	−0.53	0.69	0.62	6.39 × 10^−2^	0.0052	8.8 × 10^−2^	**4.12 × 10** ^**−2**^	1.31 × 10^−1^
ILMN_1794490	*PNMAL1*	19	8.99	8.52	−0.47	0.72	0.64	**3.14 × 10** ^**−2**^	0.0066	**4.24 × 10** ^**−2**^	**1.62 × 10** ^**−2**^	7.06 × 10^−2^
ILMN_1739393	*SELE*	1	9.88	9.29	−0.58	0.67	0.64	**4.08 × 10** ^**−2**^	0.0024	0.383	**4.65 × 10** ^**−2**^	1.31 × 10^−1^
ILMN_1730295	*SIGLEC8*	19	8.18	8.47	0.29	1.22	0.61	0.112	0.0069	**3.64 × 10** ^**−2**^	5.75 × 10^−2^	1.38 × 10^−1^
ILMN_2086105	*SPRY4*	5	12.24	12.68	0.43	1.35	0.68	**7.80 × 10** ^**−3**^	0.0079	**1.98 × 10** ^**−2**^	**1.53 × 10** ^**−2**^	7.06 × 10^−2^
ILMN_1788895	*SRD5A2*	2	11.26	10.54	−0.72	0.61	0.78	**1.00 × 10** ^**−4**^	0.0021	0.439	**1.93 × 10** ^**−4**^	3.09 × 10^−3^
ILMN_1704154	*TNFRSF19*	13	12.56	12.14	−0.42	0.75	0.65	**2.94 × 10** ^**−2**^	0.0016	0.542	8.45 × 10^−2^	1.83 × 10^−1^
ILMN_2089875	*TNFSF4*	1	8.52	8.95	0.43	1.34	0.66	**1.66 × 10** ^**−2**^	0.0149	**1.00 × 10** ^**−4**^	**2.36 × 10** ^**−2**^	8.96 × 10^−2^
ILMN_1796949	*TPX2*	20	8.53	9.13	0.60	1.52	0.65	**3.07 × 10** ^**−2**^	0.0044	0.144	**2.43 × 10** ^**−2**^	8.96 × 10^−2^
ILMN_1748124	*TSC22D3*	X	11.64	11.12	−0.53	0.69	0.77	**< 1.00 × 10** ^**−6**^	0.0073	**2.59 × 10** ^**−2**^	**3.15 × 10** ^**−5**^	1.51 × 10^−3^
ILMN_1656192	*ZNF704*	8	11.33	11.73	0.40	1.32	0.73	**4.00 × 10** ^**−4**^	0.0067	**3.91 × 10** ^**−2**^	**6.78 × 10** ^**−4**^	7.53 × 10^−3^

a Based on a *t*‐test comparing the mean transcript level between metastatic‐lethal and nonrecurrent patients.

### 3.4 Prognostic performance of validated transcripts modeled with Gleason score

**Figure 1:**
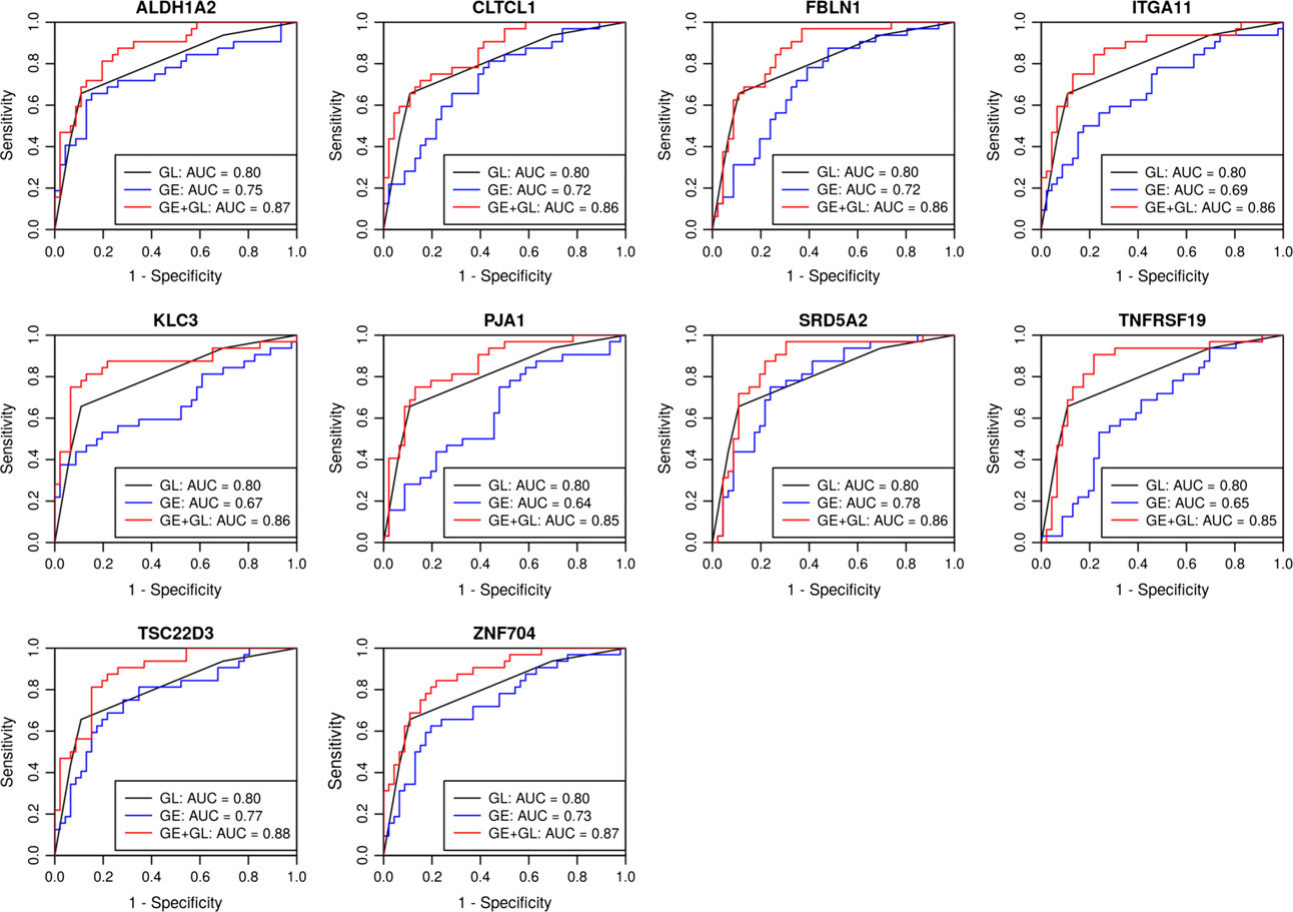
ROC curves for predicting metastatic‐lethal prostate cancer. The top 10 validated transcripts with an AUC ≥ 0.85 for the transcript + Gleason score are shown. Curves are shown for each transcript (GE = gene expression) alone, Gleason score (GL) alone, and the transcript plus Gleason score combined (GE + GL).

We next evaluated the performance of each of the 23 validated transcripts for classifying metastatic‐lethal PCa when combined with Gleason score in the EV dataset (Table 3, Fig. 1). The AUC for Gleason score alone was 0.80, which is higher than other reported studies and likely reflects the nested case–control study design in which a high percentage of patients with metastatic‐lethal outcomes had high Gleason score (8–10) tumors. Gleason score had a pAUC of 0.0084 for predicting metastatic‐lethal PCa in the EV dataset. The addition of individual transcripts to models with Gleason score alone improved all the AUCs, which ranged from 0.83 to 0.88. Fifteen of the 23 transcripts also resulted in higher pAUCs (range: 0.0107–0.0254). Likelihood ratio tests for differential gene expression between patient groups were significant (*P*‐value < 0.05) for 17 transcripts (*ALDH1A2, CENPE, CLTCL1, DPT, ITGA11*,* KLC3, PJA1, PLCL2, PNMAL1, SELE, SIGLEC8, SPRY4, TNFRSF19, TNFSF4, TPX2, TSC22D3,* and *ZNF704*), providing evidence that these biomarkers are complimentary to Gleason score for the prognostic classification of patients with PCa. Most of the validated mRNAs provided unique information about tumor aggressiveness, however, three pairs of transcripts in the validated set of 23 were correlated (*P‐*values < 0.05): *CCNA2* and *TPX2* (*r* ^2^ = 0.61); *SRD5A2* and *DPT* (*r* ^2^ = 0.56); and *SRD5A2* and *FBLN1* (*r* ^2^ = 0.73).

**Table 3:** Predictive performance of 23 validated transcripts in models combining each transcript with Gleason score in the Eastern Virginia prostate cancer testing dataset. Significant *P*‐values are shown in boldface

Transcript ID	Gene	Gleason score^a^ + transcript		*P*‐value^b^
		AUC	pAUC	
ILMN_1748538	*ALDH1A2*	0.87	0.0166	**0.006**
ILMN_1786125	*CCNA2*	0.83	0.0035	0.468
ILMN_1716279	*CENPE*	0.84	0.0107	**0.017**
ILMN_1694584	*CLTCL1*	0.86	0.0186	**0.007**
ILMN_1673843	*CST2*	0.85	0.0039	0.084
ILMN_1708107	*DPT*	0.85	0.0127	**0.027**
ILMN_1700541	*FBLN1*	0.86	0.0065	0.083
ILMN_1756358	*FBXO36*	0.84	0.0187	0.069
ILMN_2406084	*ITGA11*	0.86	0.0146	**0.024**
ILMN_1702738	*KLC3*	0.86	0.0185	**0.001**
ILMN_1661895	*PI15*	0.84	0.0079	0.115
ILMN_1734810	*PJA1*	0.86	0.0122	**0.032**
ILMN_1737025	*PLCL2*	0.84	0.0077	**0.030**
ILMN_1794490	*PNMAL1*	0.85	0.0213	**0.006**
ILMN_1739393	*SELE*	0.85	0.0160	**0.036**
ILMN_1730295	*SIGLEC8*	0.84	0.0254	**0.006**
ILMN_2086105	*SPRY4*	0.85	0.0179	**0.028**
ILMN_1788895	*SRD5A2*	0.86	0.0027	0.076
ILMN_1704154	*TNFRSF19*	0.86	0.0028	**0.016**
ILMN_2089875	*TNFSF4*	0.85	0.0157	**0.006**
ILMN_1796949	*TPX2*	0.85	0.0026	**0.039**
ILMN_1748124	*TSC22D3*	0.88	0.0180	**0.001**
ILMN_1656192	*ZNF704*	0.87	0.0171	**0.002**

^a^ For comparison with Gleason score alone: AUC = 0.80; pAUC = 0.0084. ^b^ *P*‐value for the likelihood ratio (LR) test comparing a model with Gleason score alone with a model with Gleason score and the transcript for predicting metastatic‐lethal vs. nonrecurrent prostate cancer.

### 3.5 Functional classification of gene expression panel

Functional categorization of the panel of 23 validated gene transcripts was performed using Gene Ontology, BioCarta, KEGG, NCIPID, and Reactome pathway annotations available from bioDBnet (https://biodbnet-abcc.ncifcrf.gov). These genes are included in the following broad categories: cell cycle/proliferation; cytokine/immune/inflammatory; matrix/adhesion; hormone/receptor/signal transduction; transport; and other (Table S3). Further examination using IPA (Ingenuity Pathway Analysis, www.ingenuity.com) and Upstream Regulatory Analysis indicated that expression of 11 (48%) of the 23 genes may be modulated by nuclear hormone receptors, including the *androgen receptor* (*AR*), *aryl hydrocarbon receptor* (*AHR*), *estrogen receptor 1* (*ESR1*), *glucocorticoid receptor* (*NR3C1*), and *peroxisome proliferator‐activated receptor gamma* (*PPARG*) (Fig. S2). The Fisher's exact test overlap *P*‐values were < 0.05 for all nuclear hormone receptors shown. Enrichment of the immune/inflammatory pathway was confirmed by gene set enrichment analysis (GSEA), and IPA indicated that several genes in this pathway may be regulated by the transcription factor *CEBPB* (Fig. S3).

## 4 Discussion

Our results demonstrate that gene expression levels in primary prostate tumor tissue can significantly improve upon Gleason score for distinguishing patients with clinically localized disease who will develop metastatic progression or lethal PCa from those who remain recurrence‐free for at least 5 years after radical prostatectomy. Of the 48 transcripts identified in the discovery cohort as being differentially expressed and able to discriminate between the two patient groups, with prognostic power above that of Gleason score alone, 23 were validated to predict metastatic‐lethal outcomes in an independent testing dataset.

The 23 differentially expressed transcripts reported here, which were selected using an agnostic genomewide approach, have a minimal overlap with the commercially available panels for predicting adverse patient outcomes (Table S4). Only two genes represented in our panel are also included in the Oncotype DX Prostate Cancer Assay (Klein *et al*., 2014): *SRD5A2* and *TPX2*. One gene, *TNFRSF19*, is included in the Decipher panel (Erho *et al*., 2013). There is no overlap between our validated panel of 23 mRNAs with those used to calculate the Prolaris cell cycle progression (CCP) score (Cuzick *et al*., 2011); however, four other cell cycle‐regulated genes are represented in our panel (*CCNA2*,* CENPE*,* CLTCL1*, and *TPX2*).

The genes represented by the 23 validated transcripts belong to several broad functional categories with diverse biological properties related to tumor aggressiveness, including hormone receptor signaling, adhesion, transport, inflammation, and cell cycle regulation. Eleven of these genes interact with nuclear hormone receptors [AR, AHR, ESR1, NR3C1, and PPARG (Table S3)] that regulate gene expression through ligand binding. Androgen and its receptor, AR, drive PCa development and progression (Capper *et al*., 2016), and estrogen has been shown to mediate PCa progression through the interaction with ESR1 (Mishra *et al*., 2015). The other receptors influence inflammatory response (NR3C1 and PPARG) and cell proliferation and differentiation (AHR and NR3C1).

PCa‐specific mortality results from metastasis of the primary tumor; therefore, biological drivers of metastatic progression have strong potential to predict aggressive tumor biology. Genes with functions related to this process are represented in the expression panel, including six genes in the adhesion/matrix pathway that may influence a cancer cell's ability to escape the primary tumor. Cell signaling and transport are critical functions related to cancer cell migration and establishment at a new location that are also represented by transcripts in the panel (17 and three genes, respectively). Chronic inflammation contributes to the metastatic process by providing a microenvironment that supports cancer cell growth (Gurel *et al*., 2014; Shiao *et al*., 2016). Eight of the 23 genes represented in the validated set of transcripts are in the inflammatory/immune pathway. Three of these genes were upregulated and five were downregulated in patients progressing to metastatic‐lethal outcomes. In addition, seven inflammation‐related genes are shown to be regulated by the transcription factor *CEBPB*, which functions to both promote proliferation and arrest growth in different cell types and is itself frequently dysregulated in cancer (Barakat *et al*., 2015; Willis *et al*., 2015).

Cell cycle‐regulated genes coordinate the normal cellular functions of replication, division, differentiation, and proliferation (Mosley and Keri, 2008; Whitfield *et al*., 2002, 2006). With cell cycle dysregulation, normal DNA damage response does not occur, leading to mutation accumulation, unchecked cell growth, and increased risk of metastasis. The four cell cycle‐regulated genes (*CCNA2*,* CENPE*,* CLTCL1*, and *TPX2*) in the current study were all overexpressed in patients who progressed to metastatic‐lethal PCa. Of these, *CCNA2* is of particular interest because it was also overexpressed in PCa relative to normal (benign) prostate tissue in three independent datasets, with even higher levels in metastatic samples (Grasso *et al*., 2012; Tomlins *et al*., 2007; Yu *et al*., 2004). *CCNA2* upregulation is a promising therapeutic target in part because this gene is reported to interact with a number of available cancer drugs (Gao *et al*., 2014).

The 23 transcripts confirmed in the current study were evaluated for their ability to improve upon the predictive value of Gleason score alone (AUC = 0.80), with individual transcript plus Gleason score AUC values ranging from 0.83 to 0.88. Other potential prognostic classifiers, including PSA level at diagnosis and pathological tumor stage, did not improve upon models with Gleason score only (*P‐*values > 0.05) and were therefore not included in the model. These predictive values are similar to or higher than those reported for the commercially available gene expression panels (Table S4): 0.74 for Decipher, 0.67 for Oncotype DX, and 0.88 for Prolaris (Cuzick *et al*., 2011; Erho *et al*., 2013; Klein *et al*., 2016). However, these AUC values are not directly comparable because of the differences in study design. Furthermore, the current study focused on individual transcripts, while other studies assessed the ability of transcripts combined into scores to predict the outcomes. It is important to note that there is a minimal overlap of transcripts in this study with the commercially available gene expression panels, suggesting that biomarkers from this study may provide unique biological information to improve the prognostic power of gene expression panels for distinguishing the patients at high risk of metastatic progression after radical prostatectomy.

Strengths of this study are the transcriptome‐wide approach for identifying prognostic biomarkers, the population‐based discovery cohort, the long‐term follow‐up of patients diagnosed with clinically localized disease, and the serious endpoint of metastatic‐lethal PCa. The identified transcripts were validated in an independent patient dataset, confirming their ability to improve upon Gleason score for predicting these adverse outcomes. The 23 candidates that passed the validation dataset, however, should be further validated, individually and jointly, in another independent testing dataset before they are locked down for a pivotal validation trial of clinical utility. A potential limitation is the number of patients with metastatic‐lethal PCa. However, these outcome events are rare in PCa patients diagnosed with localized tumors and treated surgically, and therefore, extended follow‐up periods are needed to accrue patients with metastatic progression. PCa is clinically and biologically heterogeneous, so a combination of biomarkers that capture a range of disease‐related biological functions will likely perform better than individual markers. Due to concerns about overfitting the data, we did not combine the 23 transcripts into a prognostic score. Future work in other independent patient cohorts is needed to combine the transcripts into a score, with the goal of improving prognostic power to predict tumor aggressiveness.

In conclusion, we identified and validated 23 genes with differential expression profiles that improve upon Gleason score for distinguishing patients who progress to metastatic‐lethal PCa from those who remain recurrence‐free for five or more years after radical prostatectomy. These genes represent diverse biological pathways related to tumor aggressiveness. Several of these are known PCa genes, but a number of them have not previously been described as playing a role in this disease and its propensity to metastasize. The gene expression biomarkers identified here have potential clinical utility for identifying the subset of patients that would benefit from closer surveillance and adjuvant therapy.

## Author contributions

JS, ZF and JF conceived and designed the project; JW, AC, MB, DT, RL, DL, EO and JF helped acquire or generate the data; SK helped with data management; RR, SZ, IC, CG, MG, AL, PN, ZF and JS analyzed and/or helped interpret the data; RR, SZ, ZF and JS drafted the manuscript; all authors read and critically revised the manuscript for intellectual content and approved the final manuscript.

## Supporting information


**Fig. S1.** Heat map of 23 validated differentially expressed transcripts.
**Fig. S2.** Ingenuity Pathway Analysis upstream regulator analysis.
**Fig. S3.** Ingenuity Pathway Analysis network of transcription factor *CEBPB*.

**Table S1.** Top‐ranked 48 gene transcripts for stratifying metastatic‐lethal vs. nonrecurrent prostate cancer.
**Table S2.** Number of times the 48 transcripts were selected.
**Table S3.** Functional classification of 23 genes represented by the validated transcripts.
**Table S4.** Summary of gene expression panels to predict prostate cancer prognosis.
