# Supplementary material for: Gene expression panel predicts metastatic‐lethal prostate cancer outcomes in men diagnosed with clinically localized prostate cancer
Source: Mol Oncol. 2016 Oct 19;11(2):140–50. doi: 10.1002/1878-0261.12014 (PMC5510189; doi:10.1002/1878-0261.12014)
Supplement: Supplementary file 1 — Fig. S1. Heat map of 23 validated differentially expressed transcripts. Fig. S2. Ingenuity Pathway Analysis upstream regulator analysis. Fig. S3. Ingenuity Pathway Analysis network of transcription factor CEBPB. [file MOL2-11-140-s001.pdf]

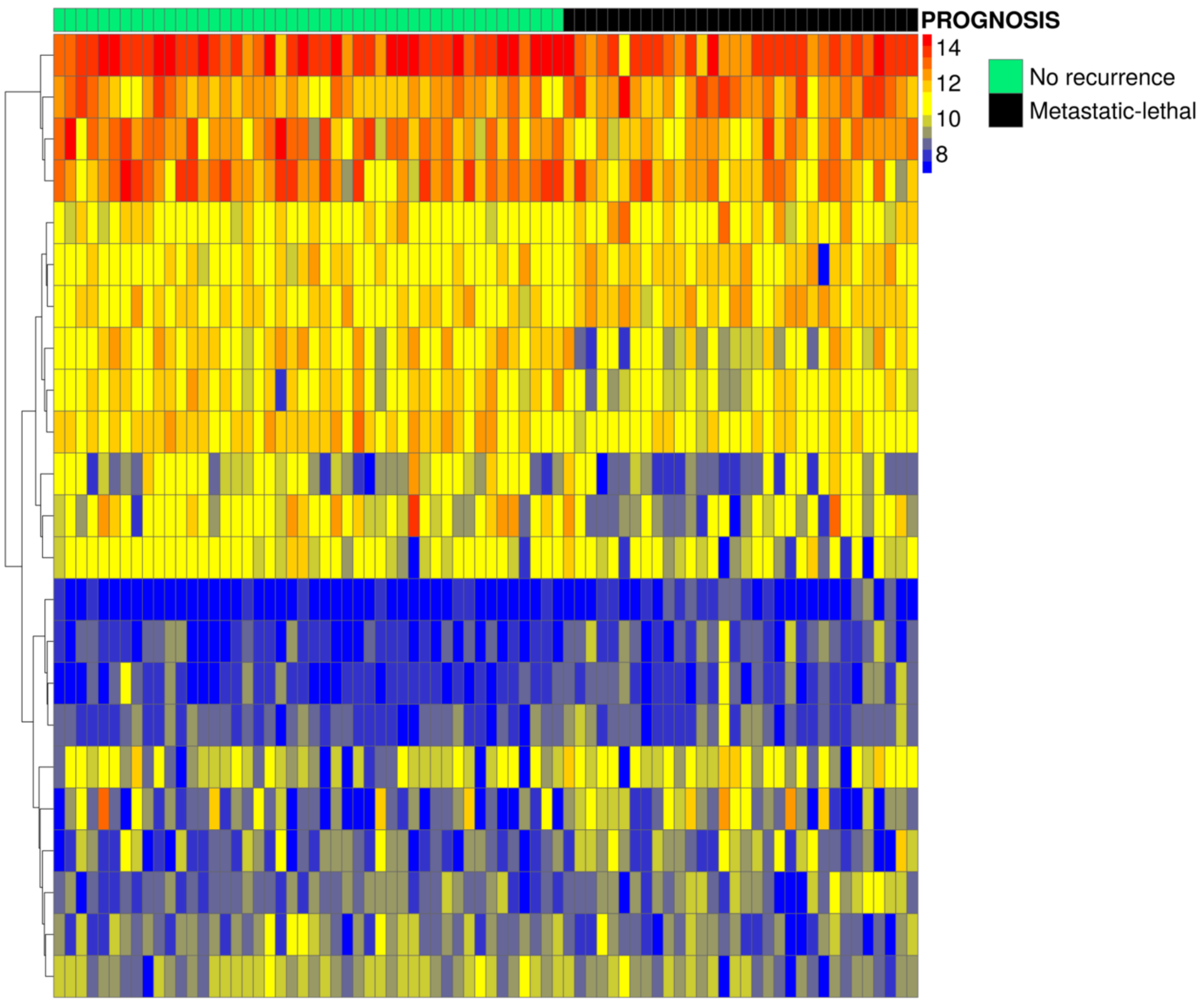

Supplemental Figure 1. Heat map of 23 validated differentially expressed transcripts in metastatic-lethal versus non-recurrent prostate cancer patients in the EV testing dataset. The rows of the heatmap are the transcripts and the columns are the tumor samples. The highest expression levels are shown in red (figure legend). There were 32 patients with metastatic-lethal progression and 46 patients with no evidence of recurrence. The rows were clustered based on Euclidean distance.

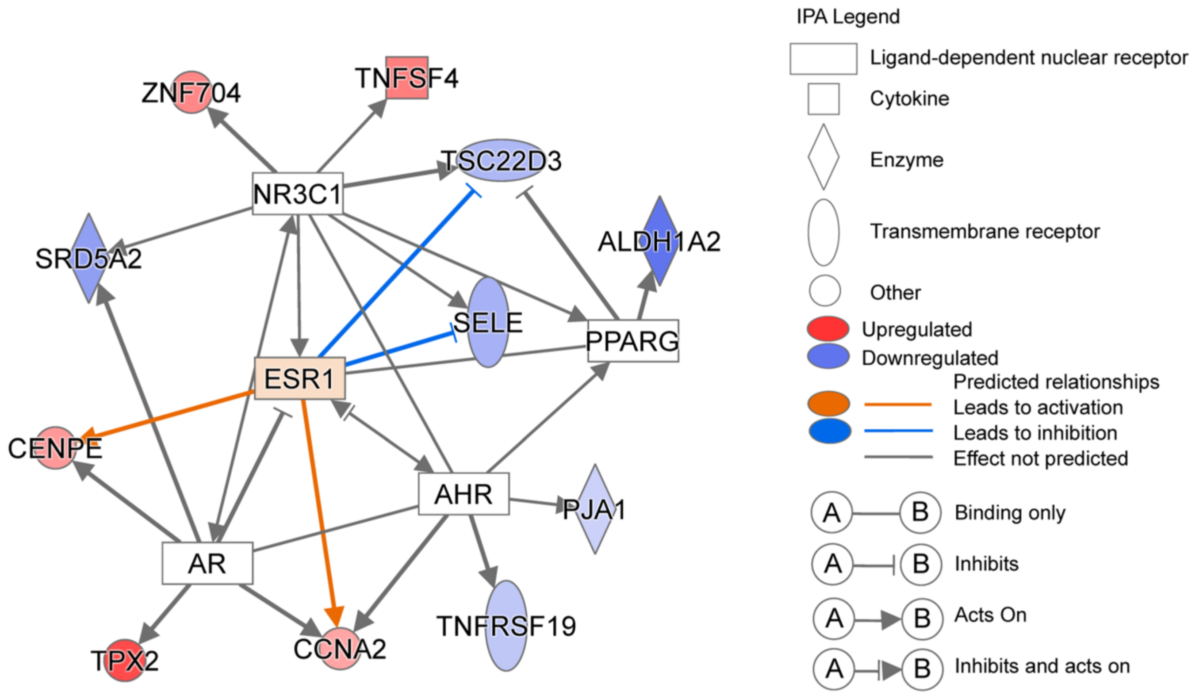

Supplemental Figure 2. Ingenuity Pathway Analysis upstream regulator analysis identified targets of nuclear hormone receptors in the gene expression panel. Expression of eleven of the 23 genes in the panel may be modulated by ligand-dependent nuclear receptors.

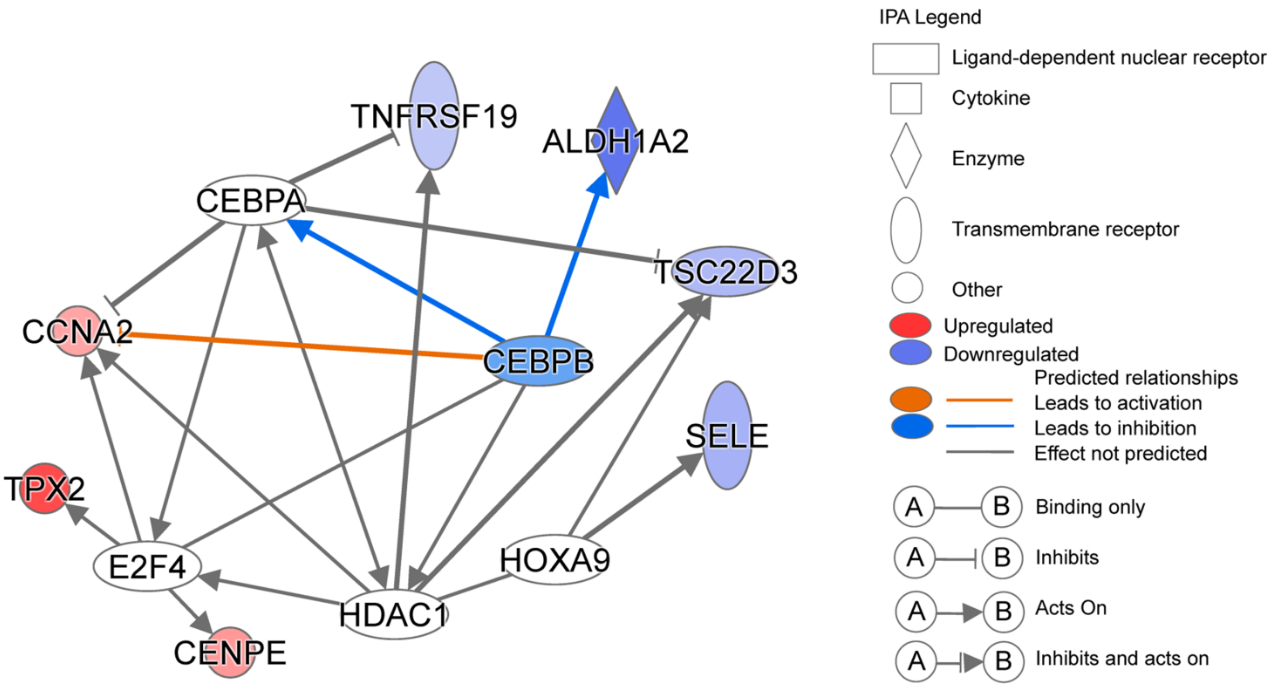

Supplemental Figure 3. Ingenuity Pathway Analysis network of transcription factor *CEBPB* and its regulation of the expression of immune/inflammatory genes. Expression of several of the 23 genes in the panel may be regulated by *CEBPB*.
