# Supplementary material for: Gene expression panel predicts metastatic‐lethal prostate cancer outcomes in men diagnosed with clinically localized prostate cancer
Source: Mol Oncol. 2016 Oct 19;11(2):140–50. doi: 10.1002/1878-0261.12014 (PMC5510189; doi:10.1002/1878-0261.12014)
Supplement: Supplementary file 2 — Table S1. Top‐ranked 48 gene transcripts for stratifying metastatic‐lethal vs. nonrecurrent prostate cancer. Table S2. Number of times the 48 transcripts were selected. Table S3. Functional classification of 23 genes represented by the validated transcripts. Table S4. Summary of gene expression panels to predict prostate cancer prognosis. [file MOL2-11-140-s002.docx]

Supplemental Table 1. Top-ranked 48 gene transcripts for stratifying metastatic-lethal vs. non-recurrent prostate cancer patients in the Fred Hutchinson (discovery) cohort

| Transcript ID | Gene | Chr. | Mean expression non-recurrence | Mean expression metastatic-lethal | Difference in mean expression | Fold-change | *P*-value^a^ | AUC | pAUC |
| --- | --- | --- | --- | --- | --- | --- | --- | --- | --- |
| ILMN_1812070 | *ABCB1* | 7 | 9.78 | 9.01 | -0.77 | 0.59 | 6.61x10^-4^ | 0.74 | 0.0186 |
| **ILMN_1748538** | *ALDH1A2* | 15 | 12.06 | 10.85 | -1.22 | 0.43 | 7.67x10^-6^ | 0.81 | 0.0112 |
| ILMN_1707277 | *CCDC144A* | 17 | 10.79 | 9.96 | -0.84 | 0.56 | 3.89x10^-3^ | 0.68 | 0.0099 |
| **ILMN_1786125** | *CCNA2* | 4 | 10.87 | 11.53 | 0.65 | 1.57 | 1.31x10^-5^ | 0.81 | 0.0125 |
| ILMN_1766675 | *CDH6* | 5 | 10.62 | 11.05 | 0.43 | 1.35 | 5.51x10^-2^ | 0.62 | 0.0100 |
| **ILMN_1716279** | *CENPE* | 4 | 8.40 | 9.16 | 0.75 | 1.68 | 1.75x10^-3^ | 0.71 | 0.0135 |
| ILMN_1796939 | *CHAT* | 10 | 7.49 | 7.65 | 0.15 | 1.11 | 1.01x10^-2^ | 0.65 | 0.0123 |
| ILMN_1679638 | *CHN1* | 2 | 7.56 | 7.81 | 0.26 | 1.19 | 3.68x10^-3^ | 0.68 | 0.0105 |
| **ILMN_1694584** | *CLTCL1* | 22 | 8.24 | 8.82 | 0.58 | 1.50 | 3.23x10^-3^ | 0.70 | 0.0114 |
| **ILMN_1673843** | *CST2* | 20 | 9.19 | 10.54 | 1.35 | 2.55 | 7.31x10^-5^ | 0.76 | 0.0086 |
| ILMN_1697914 | *DGKB* | 7 | 7.65 | 7.92 | 0.26 | 1.20 | 5.04x10^-2^ | 0.58 | 0.0106 |
| **ILMN_1708107** | *DPT* | 1 | 11.63 | 10.29 | -1.35 | 0.39 | 3.35x10^-6^ | 0.82 | 0.0101 |
| ILMN_1798706 | *EBI2* | 13 | 10.90 | 9.91 | -0.99 | 0.50 | 1.12x10^-3^ | 0.72 | 0.0107 |
| ILMN_1761551 | *FAT4* | 4 | 9.48 | 9.10 | -0.37 | 0.77 | 2.88x10^-2^ | 0.66 | 0.0114 |
| **ILMN_1700541** | *FBLN1* | 22 | 14.48 | 13.67 | -0.81 | 0.57 | 1.04x10^-5^ | 0.80 | 0.0149 |
| **ILMN_1756358** | *FBXO36* | 2 | 11.31 | 11.48 | 0.17 | 1.13 | 0.227 | 0.63 | 0.0111 |
| ILMN_1751607 | *FOSB* | 19 | 14.69 | 14.36 | -0.33 | 0.79 | 1.23x10^-3^ | 0.76 | 0.0045 |
| ILMN_1659678 | *GABRA2* | 4 | 8.24 | 8.98 | 0.74 | 1.67 | 3.83x10^-2^ | 0.56 | 0.0132 |
| ILMN_1751596 | *HIVEP3* | 1 | 9.83 | 9.31 | -0.52 | 0.70 | 8.90x10^-3^ | 0.65 | 0.0112 |
| **ILMN_2406084** | *ITGA11* | 15 | 10.06 | 10.77 | 0.71 | 1.64 | 2.53x10^-4^ | 0.73 | 0.0100 |
| ILMN_1715133 | *IVNS1ABP* | 1 | 11.09 | 10.04 | -1.05 | 0.48 | 3.60x10^-4^ | 0.79 | 0.0089 |
| **ILMN_1702738** | *KLC3* | 19 | 7.71 | 7.96 | 0.24 | 1.18 | 9.41x10^-2^ | 0.54 | 0.0112 |
| ILMN_2172497 | *LPPR4* | 1 | 7.69 | 7.96 | 0.28 | 1.21 | 7.92x10^-3^ | 0.65 | 0.0103 |
| ILMN_1760441 | *MRPS5* | 2 | 10.82 | 10.56 | -0.26 | 0.84 | 0.132 | 0.54 | 0.0118 |
| ILMN_1691156 | *MT1A* | 16 | 8.14 | 7.57 | -0.57 | 0.67 | 2.90x10^-9^ | 0.76 | 0.0099 |
| ILMN_1782305 | *NR4A2* | 2 | 11.49 | 10.33 | -1.16 | 0.45 | 8.00x10^-6^ | 0.80 | 0.0131 |
| ILMN_2406724 | *NUF2* | 1 | 7.50 | 7.63 | 0.14 | 1.10 | 3.54x10^-3^ | 0.70 | 0.0122 |
| ILMN_1721578 | *OR5P2* | 11 | 7.72 | 7.99 | 0.26 | 1.20 | 3.28x10^-2^ | 0.61 | 0.0100 |
| ILMN_2319588 | *OSGIN1* | 16 | 8.86 | 9.57 | 0.72 | 1.64 | 2.88x10^-5^ | 0.76 | 0.0055 |
| **ILMN_1661895** | *PI15* | 8 | 12.95 | 12.11 | -0.84 | 0.56 | 5.98x10^-4^ | 0.75 | 0.0071 |
| **ILMN_1734810** | *PJA1* | X | 9.14 | 8.60 | -0.54 | 0.69 | 2.00x10^-3^ | 0.73 | 0.0119 |
| **ILMN_1737025** | *PLCL2* | 3 | 11.01 | 9.83 | -1.19 | 0.44 | 2.04x10^-5^ | 0.79 | 0.0117 |
| **ILMN_1794490** | *PNMAL1* | 19 | 9.48 | 8.77 | -0.72 | 0.61 | 2.19x10^-4^ | 0.73 | 0.0119 |
| ILMN_1738675 | *PTPN6* | 12 | 11.34 | 10.82 | -0.52 | 0.70 | 7.53x10^-3^ | 0.68 | 0.0114 |
| **ILMN_1739393** | *SELE* | 1 | 9.90 | 8.87 | -1.04 | 0.49 | 3.15x10^-8^ | 0.80 | 0.0058 |
| ILMN_1795341 | *SFRS1* | 17 | 11.90 | 11.64 | -0.26 | 0.84 | 3.27x10^-3^ | 0.70 | 0.0112 |
| **ILMN_1730295** | *SIGLEC8* | 19 | 8.47 | 9.04 | 0.58 | 1.49 | 5.77x10^-5^ | 0.77 | 0.0092 |
| ILMN_1732489 | *SLC10A7* | 4 | 12.27 | 12.88 | 0.61 | 1.53 | 5.05x10^-4^ | 0.72 | 0.0127 |
| ILMN_1806979 | *SLCO1A2* | 12 | 7.52 | 7.70 | 0.18 | 1.13 | 7.04x10^-3^ | 0.67 | 0.0132 |
| **ILMN_2086105** | *SPRY4* | 5 | 12.29 | 12.79 | 0.50 | 1.41 | 9.88x10^-4^ | 0.69 | 0.0098 |
| **ILMN_1788895** | *SRD5A2* | 2 | 11.20 | 10.48 | -0.72 | 0.61 | 7.43x10^-4^ | 0.72 | 0.0145 |
| ILMN_1765578 | *TIPARP* | 3 | 9.30 | 8.56 | -0.74 | 0.60 | 5.58x10^-8^ | 0.79 | 0.0036 |
| **ILMN_1704154** | *TNFRSF19* | 13 | 12.85 | 11.82 | -1.02 | 0.49 | 9.84x10^-6^ | 0.80 | 0.0096 |
| **ILMN_2089875** | *TNFSF4* | 1 | 9.17 | 9.54 | 0.36 | 1.29 | 2.10x10^-2^ | 0.63 | 0.0104 |
| ILMN_1729318 | *TOR1AIP1* | 1 | 13.26 | 13.40 | 0.14 | 1.10 | 0.140 | 0.62 | 0.0111 |
| **ILMN_1796949** | *TPX2* | 20 | 8.93 | 9.81 | 0.89 | 1.85 | 2.16x10^-4^ | 0.77 | 0.0118 |
| **ILMN_1748124** | *TSC22D3* | X | 11.57 | 11.08 | -0.49 | 0.71 | 1.40x10^-2^ | 0.66 | 0.0123 |
| **ILMN_1656192** | *ZNF704* | 8 | 11.31 | 11.89 | 0.58 | 1.50 | 8.84x10^-8^ | 0.84 | 0.0139 |

^a^ Based on a t-test comparing mean gene expression level between metastatic-lethal and non-recurrent PCa patients

Transcripts that validated in the Eastern Virginia testing dataset are shown in boldface.

Supplemental Table 2. Number of times the 48 transcripts were selected for inclusion in the model (1,000 bootstraps, per criterion)^a^

| Transcript ID | Gene | AUC | pAUC | t-test *P*-value |
| --- | --- | --- | --- | --- |
| ILMN_1812070 | *ABCB1* | 12 | 43 | 10 |
| **ILMN_1748538** | *ALDH1A2* | 3 | 4 | 49 |
| ILMN_1707277 | *CCDC144A* | 15 | 44 | 51 |
| **ILMN_1786125** | *CCNA2* | 73 | 10 | 34 |
| ILMN_1766675 | *CDH6* | 10 | 44 | 44 |
| **ILMN_1716279** | *CENPE* | 13 | 15 | 40 |
| ILMN_1796939 | *CHAT* | 4 | 10 | 49 |
| ILMN_1679638 | *CHN1* | 26 | 5 | 47 |
| **ILMN_1694584** | *CLTCL1* | 75 | 61 | 175 |
| **ILMN_1673843** | *CST2* | 16 | 67 | 116 |
| ILMN_1697914 | *DGKB* | 3 | 38 | 43 |
| **ILMN_1708107** | *DPT* | 13 | 3 | 43 |
| ILMN_1798706 | *EBI2* | 8 | 22 | 45 |
| ILMN_1761551 | *FAT4* | 1 | 13 | 136 |
| **ILMN_1700541** | *FBLN1* | 6 | 35 | 74 |
| **ILMN_1756358** | *FBXO36* | 24 | 19 | 55 |
| ILMN_1751607 | *FOSB* | 15 | 17 | 96 |
| ILMN_1659678 | *GABRA2* | 4 | 145 | 54 |
| ILMN_1751596 | *HIVEP3* | 14 | 27 | 45 |
| **ILMN_2406084** | *ITGA11* | 51 | 21 | 89 |
| ILMN_1715133 | *IVNS1ABP* | 91 | 2 | 25 |
| **ILMN_1702738** | *KLC3* | 2 | 33 | 81 |
| ILMN_2172497 | *LPPR4* | 200 | 95 | 426 |
| ILMN_1760441 | *MRPS5* | 25 | 76 | 142 |
| ILMN_1691156 | *MT1A* | 79 | 21 | 13 |
| ILMN_1782305 | *NR4A2* | 53 | 8 | 110 |
| ILMN_2406724 | *NUF2* | 50 | 73 | 87 |
| ILMN_1721578 | *OR5P2* | 5 | 77 | 95 |
| ILMN_2319588 | *OSGIN1* | 17 | 8 | 75 |
| **ILMN_1661895** | *PI15* | 49 | 3 | 52 |
| **ILMN_1734810** | *PJA1* | 9 | 27 | 58 |
| **ILMN_1737025** | *PLCL2* | 1 | 9 | 75 |
| **ILMN_1794490** | *PNMAL1* | 0 | 42 | 0 |
| ILMN_1738675 | *PTPN6* | 4 | 55 | 14 |
| **ILMN_1739393** | *SELE* | 89 | 56 | 92 |
| ILMN_1795341 | *SFRS1* | 41 | 10 | 40 |
| **ILMN_1730295** | *SIGLEC8* | 36 | 65 | 169 |
| ILMN_1732489 | *SLC10A7* | 6 | 37 | 48 |
| ILMN_1806979 | *SLCO1A2* | 29 | 8 | 42 |
| **ILMN_2086105** | *SPRY4* | 23 | 46 | 46 |
| **ILMN_1788895** | *SRD5A2* | 9 | 47 | 28 |
| ILMN_1765578 | *TIPARP* | 102 | 5 | 11 |
| **ILMN_1704154** | *TNFRSF19* | 61 | 43 | 130 |
| **ILMN_2089875** | *TNFSF4* | 10 | 29 | 51 |
| ILMN_1729318 | *TOR1AIP1* | 68 | 38 | 156 |
| **ILMN_1796949** | *TPX2* | 35 | 46 | 75 |
| **ILMN_1748124** | *TSC22D3* | 8 | 17 | 48 |
| **ILMN_1656192** | *ZNF704* | 271 | 75 | 126 |

^a^ Transcripts shown in boldface were validated in the Eastern Virginia dataset.

Supplemental Table 3. Functional classification of 23 genes represented by the validated transcripts according to Gene Ontology, Biocarta, KEGG, NCIPID, and Reactome pathway databases

| Gene Symbol | cell cycle / proliferation | cytokine / immune / inflammatory | matrix / adhesion | hormone / receptor / signaling | transport | other |
| --- | --- | --- | --- | --- | --- | --- |
| *CENPE* |  |  |  |  |  |  |
| *CLTCL1* |  |  |  |  |  |  |
| *CCNA2* |  |  |  |  |  |  |
| *TPX2* |  |  |  |  |  |  |
| *SELE* |  |  |  |  |  |  |
| *TNFRSF19* |  |  |  |  |  |  |
| *PLCL2* |  |  |  |  |  |  |
| *ALDH1A2* |  |  |  |  |  |  |
| *TNFSF4* |  |  |  |  |  |  |
| *PJA1* |  |  |  |  |  |  |
| *KLC3* |  |  |  |  |  |  |
| *SIGLEC8* |  |  |  |  |  |  |
| *SPRY4* |  |  |  |  |  |  |
| *ITGA11* |  |  |  |  |  |  |
| *FBLN1* |  |  |  |  |  |  |
| *DPT* |  |  |  |  |  |  |
| *TSC22D3* |  |  |  |  |  |  |
| *SRD5A2* |  |  |  |  |  |  |
| *ZNF704* |  |  |  |  |  |  |
| *PI15* |  |  |  |  |  |  |
| *CST2* |  |  |  |  |  |  |
| *FBXO36* |  |  |  |  |  |  |
| *PNMAL1* |  |  |  |  |  |  |

Supplemental Table 4. Summary of gene expression panels designed to predict prostate cancer prognosis

|  | | |  |  |  |  |  |
| --- | --- | --- | --- | --- | --- | --- | --- |
| Study | Patient population | Number of events | Description | AUC for model | Biomarkers/transcripts/  genes evaluated | Gene transcripts in the panel | Genes overlapping with current study panel |
| Nakagawa et al., 2008 | U.S. RP patients:  Training: n=391  Validation: n=205 | Mets/PCa death: n=133  Mets/PCa death: n=67 | Predict systemic progression after PSA recurrence^a^ | AUC = 0.86^b^  -- | 1021 | 17 | 0 |
| Cuzick et al., 2011 | U.K. TURP patients: n=337 | PCa death: n=68 | Prolaris (Myriad Genetics, Inc.) Cell cycle progression (CCP) score to predict PCa aggressiveness^c^ | AUC = 0.88^d^ | 126 | 46 (31 cell cycle progression genes +15 reference genes) | 0 |
| Penney et al., 2011 | Swedish TURP patients^e^  Training: n=358  U.S. RP or TURP patients  Validation: n=109 | PCa death: n=171  PCa death: n=30 | Gleason expression panel to predict PCa death^f^ | AUC = 0.91^g^  AUC = 0.94^g^ | 6100 | 157 | 0 |
| Erho et al., 2013 | U.S. RP patients:  Training: n=359  Validation: n=186 | Mets/PCa death: n=129  Mets/PCa death: n=63 | Decipher Prostate Cancer Classifier (GenomeDx) to predict early metastasis^h^ | AUC = 0.91^i^  AUC = 0.74^i^ | 1.4 million^j^ | 22 | 1 (*TNFRSF19*) |
| Klein et al., 2014 | U.S. RP patients  Training: n=441  U.S. Biopsy patients^k^  Training: n=167  U.S. Biopsy patients^k^  Validation: n=395 | PCa death n=45  Aggressive PCa:^l^ n=58  Aggressive PCa:^l^ n=124 | Oncotype DX Prostate Cancer Assay (Genomic Health) to predict PCa aggressiveness^m^ | AUC = 0.67^n^ | 732 | 17 (12 genes + 5 reference genes) | 2 (*SRD5A2*; *TPX2*) |
| Current study | U.S. RP patients:  Training: n=305  Validation: n=78 | Mets/PCa death: n=27  Mets/PCa death: n=32 | Panel to predict metastatic-lethal PCa after RP | AUC = 0.83 – 0.88^o^ | 26,051 | 23 | -- |

Mets=metastatic; PCa=prostate cancer; RP=radical prostatectomy; TURP=transurethral resection of the prostate

^a^Systemic progression defined as a positive bone or CT scan within 5 years of rising PSA (post-RP PSA ≥0.20 ng/mL, with next PSA ≥0.05 higher or initiation of treatment for PSA recurrence).

^b^AUC for 17 gene/probe model plus clinical variables (revised Gleason score and pathologic stage) for training set. AUC = 0.85 for 17 gene/probe model alone for training set.

^c^For TURP patients the panel was used to predict PCa death.

^d^AUC for CCP score combined with clinical variables (PSA and Gleason score). AUC = 0.81 for clinical variables alone.

^e^Swedish cohort consists of watchful waiting patients.

^f^Expression panel developed from patients with high (≥8) vs. low (≤6) Gleason score tumors, and used to predict PCa death in patients with intermediate Gleason score (7).

^g^AUCs for 157-gene model for discriminating between high (≥8) vs. low (≤6) Gleason score tumors in training and validation sets. AUC = 0.60 (95% 0.53-0.68) for 157-gene model for distinguishing between intermediate Gleason score tumors (7 = 3+4) vs. (7 = 4+3). The 157-gene signature significantly improved prediction of lethal disease for patients with intermediate Gleason score tumors, beyond that of Gleason score 7 = 3+4 vs. 7 = 4+3 alone (P = 0.006)

^h^Expression panel developed to predict early clinical metastasis (confirmed by bone or CT scan) within 5 years of biochemical recurrence (post-RP PSA ≥0.20 ng/mL, with next PSA ≥0.05 higher).

^i^AUCs for model with 22 genes and clinical variables (pathological Gleason score, pre-operative PSA, positive surgical margins, seminal vesicle invasion, extra-capsular extension, and lymph node involvement) for training and validation sets.

^j^Evaluated 1.4 million RNA features (including non-coding RNAs and ~22,000 protein-coding genes).

^k^Patients had biopsies and were candidates for active surveillance, but elected to have RP within 6 months of biopsy. RNA analysis was performed on biopsy samples, and RP samples were used to determine Gleason patterns and pathologic T stage.

^l^Aggressive PCa was defined as primary Gleason pattern 4 or any pattern 5, and/or non-organ-confined disease (pathologic stage pT3), at prostatectomy.

^m^Expression panel developed to predict aggressive PCa (defined as primary Gleason pattern 4 or any pattern 5, and/or non-organ-confined disease (pathologic stage pT3)) using biopsy samples from clinically localized tumors.

^n^AUC is for predicting favorable pathology [no evidence of high grade disease (defined as primary Gleason pattern 4 or any pattern 5) and no non-organ-confined disease (pathologic stage pT3), at prostatectomy] for the validation cohort.

^o^Range of AUCs for models with individual transcript combined with Gleason score for predicting metastatic progression or lethal PCa in validation set.
